# Prioritising modifiable risk factors for self-management of urinary incontinence in older men: integrating expert consensus with patient readiness

**DOI:** 10.1093/ageing/afag270

**Published:** 2026-09-14

**Authors:** Olawunmi Olagundoye, Thomas F Monaghan, William Gibson, Adrian Wagg

**Affiliations:** Division of Geriatric Medicine, Department of Medicine, Faculty of Medicine & Dentistry, College of Health Sciences, University of Alberta, Edmonton, Alberta, Canada; Department of Urology, The University of Texas Southwestern Medical Center Medical School, Dallas, TX, USA; Division of Geriatric Medicine, Department of Medicine, Faculty of Medicine & Dentistry, College of Health Sciences, University of Alberta, Edmonton, Alberta, Canada; Division of Geriatric Medicine, Department of Medicine, Faculty of Medicine & Dentistry, College of Health Sciences, University of Alberta, Edmonton, Alberta, Canada

**Keywords:** urinary incontinence, aged, male, risk factors, self-care, older people

## Abstract

**Background:**

Urinary incontinence (UI) affects up to one-third of men aged ≥65 years, yet evidence to guide the selection of targets for self-management interventions remains limited. Although many contributing risk factors are potentially modifiable, little is known about which are considered clinically important by experts and are also viewed by older men as feasible and worthwhile to address. This study combined expert consensus with older men’s perspectives to identify candidate priorities for future self-management intervention development.

**Methods:**

We conducted a sequential multi-methods study: an international Delphi survey with continence care experts followed by a cross-sectional survey of community-dwelling men aged ≥65 years with UI in Alberta, Canada. Experts rated the clinical importance and modifiability of evidence-informed risk factors, with consensus defined as ≥75% agreement. Older men reported willingness and perceived capability to modify expert-identified factors and completed the Geriatric Self-Efficacy Index for Urinary Incontinence (GSE-UI).

**Results:**

Experts (*n* = 32 in Round 1; *n* = 20 in the final round) reached consensus on 19 clinically important and modifiable risk factors, spanning medical (obesity, constipation, diabetes) and behavioural (smoking, alcohol, caffeine) domains. Older men (*n* = 212, mean age 77.6 years) reported moderate self-efficacy (overall GSE-UI—51.0 ± 35.9), with lower confidence in socially or physically challenging situations.

**Conclusion:**

Integrating expert consensus with patient-reported willingness and perceived capability identified nocturia/nocturnal polyuria, insomnia/sleep disorder, increased body mass index (BMI)/obesity and anxiety as candidate priorities for future self-management intervention development. Increased BMI/obesity and anxiety exceeded the thresholds for prevalence, willingness and perceived capability, whereas nocturia/nocturnal polyuria and insomnia/sleep disorder exceeded the thresholds for prevalence and willingness but not perceived capability. Integrating expert consensus with older men’s willingness and perceived capability provides an empirical approach to prioritising candidate intervention targets rather than relying on clinical importance alone. These findings provide a rationale for designing and evaluating future self-management interventions that better align with both clinical priorities and patient readiness.

## Key Points

- Expert consensus identified 19 clinically important and modifiable risk factors for urinary incontinence in older men.
- Older men reported moderate UI self-efficacy, with variable willingness and perceived capability to modify risk factors.
- Integrating expert and patient perspectives identified nocturia, obesity, insomnia and anxiety as candidate targets.

## Introduction

Urinary incontinence (UI) affects up to one in three men aged 65 years and older and has far-reaching physical, psychological and social consequences [1]. Among older men, UI is associated with increased risks of institutionalisation, falls, depression, poorer quality of life and increased healthcare utilisation, highlighting its importance as a late-life health concern [2, 3]. Despite this, UI in men remains under-recognised and undertreated, partly due to stigma and the misconception of UI as a predominantly female condition [4]. Many men delay seeking care until symptoms become severe, or avoid seeking treatment altogether, highlighting the need for more accessible, self-directed management strategies [4–6]. The cause of UI in older men is multifactorial; contributing factors include medical comorbidities, bowel dysfunction, sleep disturbances, lower urinary tract symptoms (LUTS), functional limitations and lifestyle behaviours [7]. Although several of these factors are potentially modifiable, existing conservative and self-management interventions have largely been extrapolated from women and younger men with LUTS [8]. Little is known about which risk factors older men themselves feel ready and able to modify.

Self-management, an evidence-informed approach widely used for other chronic conditions, encourages individuals to identify problems, set goals and take active steps in managing symptoms [9, 10]. Effective self-management requires clinical relevance and behavioural feasibility [11]. While expert opinion and clinical evidence help identify potentially modifiable risk factors, successful intervention development also requires understanding whether intended users perceive these factors as relevant, feasible and worthwhile to address [9–12]. Integrating clinical priorities with patient perspectives may help identify intervention targets that are both clinically important and behaviourally acceptable.

Self-efficacy refers to confidence in one’s ability to perform the behaviours required to achieve a desired outcome [13]. In the context of UI, it is defined as confidence in one’s ability to manage symptoms and related challenges in specific situations, rather than being limited to modifying contributing risk factors [14]. In contrast, perceived capability in this study refers to participants’ perceived ability to modify contributing risk factors. Together, willingness and perceived capability reflect readiness for behaviour change. Among older adults, higher continence self-efficacy has been associated with improved continence outcomes and adherence to behavioural interventions [14].

Understanding both is essential for designing effective, patient-centred interventions.

As part of a project to create an evidence-informed self-management programme for older men with UI, we conducted a sequential, multi-method study integrating expert consensus and patient-reported behavioural readiness with the following objectives:

(1) Identify UI risk factors in older men considered clinically important and modifiable by experts.

(2) Assess older men’s self-efficacy for managing UI.

(3) Determine which modifiable risk factors older men are most willing and perceive themselves capable of changing.

This work builds on our previously published scoping review [7] by moving beyond identifying potentially modifiable risk factors to identifying priorities for future intervention development when both expert consensus and patient-reported readiness are considered.

Accordingly, this study was designed as a prioritisation exercise for future self-management intervention development, potentially allowing a future assessment of intervention effectiveness. By integrating expert-identified modifiable UI risk factors with older men’s willingness and perceived capability to modify those factors, we sought to identify candidate intervention targets for future evaluation.

## Materials and methods

### Study design

A sequential multi-method design was used, consisting of (i) a Delphi study with continence care experts and (ii) a cross-sectional survey of older men with UI. Reporting adhered to the Guidance on Conducting and Reporting Delphi Studies and the Checklist for Reporting Of Survey Studies checklists [15, 16]. The study was approved by the institutional health research ethics board (Approval No. Pro00134947). All participants provided informed consent, and additional permissions were obtained from participating organisations.

### Phase 1

#### Delphi panel

##### Participants and sampling

Healthcare professionals with expertise in continence care were recruited internationally via snowball sampling through professional organisations and literature searches. An international Delphi approach was used to obtain informed expert consensus across diverse clinical backgrounds and settings. This is particularly relevant in UI, where diagnostic thresholds, management strategies and care pathways vary across regions and specialties. The Delphi method prioritises iterative consensus-building among diverse expert perspectives rather than statistical representativeness of any specific healthcare system or population. The panel included urologists, geriatricians, family physicians and nurse practitioners, reflecting clinicians primarily involved in the clinical assessment and medical management of UI in older men. Attrition across rounds is a recognised feature of Delphi processes; however, predefined consensus thresholds were met in each round, and the panel retained sufficient disciplinary diversity across iterations.

An evidence-derived list of 47 behavioural, physiological, age-related, demographic, medical, environmental, genetic and other UI risk factors was derived from a prior scoping review [7]. These factors were presented to panellists, who rated the importance of each factor in contributing to UI in older men, as well as their opinion on the modifiability of each factor. Consensus was defined, a priori, as ≥75% agreement and rounds required ≥70% participant completion to proceed [15]. After each round, anonymised group feedback and qualitative summaries were provided to allow response revision [17]. The Delphi process concluded after four rounds (Supplementary Appendix S1).

Thirty-two healthcare professionals participated in Round 1 and 20 in Round 4, providing an adequate sample for a heterogeneous panel of experts [18].

### Phase 2

#### Older men’s survey

##### Participants and sampling

This phase was distinct from the international Delphi process. Using Delphi outcomes, a survey, conducted across Alberta, Canada, assessed older men’s willingness and perceived capability to modify expert-identified risk factors. Community-dwelling men aged ≥65 years with self-reported UI were eligible. Eligibility was confirmed using the Sandvik UI Severity Index [19]. Based on a 95% confidence level, a 5% margin of error and a 9.2% prevalence of UI in community-dwelling older men, a minimum sample size of 128 participants was calculated (Canadian Community Health Survey—Healthy Aging) [20]. This exceeded the minimum sample size for stable estimation of Likert-scale measures and subgroup prevalence estimates. The sample size calculation was precision-based and intended to support stable estimation of prevalence and behavioural readiness measures within the study sample.

Participants were recruited through seniors’ organisations, retirement communities, religious institutions and a mailing from a comprehensive database of men with healthcare encounters related to UI identified from the database of Alberta Health Services under an established permission-to-contact agreement. Participants were recruited via convenience sampling using online and paper surveys.

### Measures

All study materials were available in English and French. French-language materials were translated by a bilingual native French speaker to support participation in Canada’s official languages. The survey was piloted with 30 men aged ≥65 years (who were not included in the main study) to establish face validity and assess reliability. Internal consistency was high (Cronbach’s α = 0.97). Participant feedback was used to refine the final survey.

Continence self-efficacy was measured using the Geriatric Self-Efficacy Index for Urinary Incontinence (GSE-UI) [21], a validated 12-item scale assessing confidence in preventing urinary leakage across a range of everyday situations, including social, physical and home-based contexts. Each item is rated on a 0–10 scale, with total scores ranging from 0 to 120; higher scores indicate greater confidence in managing incontinence. The overall score is the sum of all item scores. The scale has been previously used to identify areas of low self-efficacy and guide self-management interventions.

In this study, ‘willingness’ was operationalised as self-reported motivation to change a given risk factor, while ‘capability’ was assessed as participants’ perceived ability to implement that change. Although these measures align conceptually with the motivation and capability components of the Capability, Opportunity, Motivation–Behaviour (COM-B) model [22], they reflect subjective perceptions rather than objective capability or observed behaviour. As self-reported measures, they may be influenced by individual, social and contextual factors and are indicators of readiness to change rather than measures of actual behaviour change or adherence [22].

Behavioural readiness was assessed for each expert-identified modifiable risk factor using a 4-point Likert scale. Willingness was rated from 0 (‘I do not want to make a change’) to 3 (‘I definitely want to make a change’), and capability was rated from 0 (‘I am definitely not able to make a change’) to 3 (‘I am definitely able to make a change’). A fifth response option, ‘Does not apply to me’, was excluded from scoring and used only to determine factor prevalence, calculated as the proportion of respondents for whom the factor was applicable (i.e. 100% minus the proportion selecting ‘Does not apply to me’). Missing responses (‘no response’) were excluded from factor-specific analyses; no data imputation was performed. For each risk factor, willingness and capability scores were calculated as the mean of responses among participants for whom the factor was applicable.

### Statistical analysis

Continuous variables are reported as mean ± SD, categorical as frequencies and percentages.

Delphi panel data were pooled for analysis, and no between-country comparisons were performed, consistent with the study aim of identifying broadly relevant modifiable risk factors rather than cultural differences.

Likert-type responses were summarised using means to facilitate relative comparisons across factors, an approach supported for multi-item Likert scales in exploratory analyses [23]. Exploratory associations between self-efficacy, willingness and capability were examined using Spearman’s rank correlations, to describe relationships between constructs rather than test hypotheses [23]. Risk factor prioritisation used a quadrantic framework based on factor prevalence and mean willingness and perceived capability scores. As no validated thresholds exist for willingness or perceived capability to modify UI-related risk factors, empirical thresholds were derived as the mean of the 19 factor-specific willingness scores, capability scores and prevalence. This framework was intended as an exploratory prioritisation tool rather than a statistical classification method.

Given the exploratory nature of the study, analyses were descriptive and no adjustment for multiple comparisons was applied, as the aim was to characterise patterns of association.

Online data were collected directly via the secure REDCap electronic data capture tools hosted at the study institution [24]. Paper response data were transferred into the REDCap system to consolidate all data.

Delphi responses were analysed for consensus, with qualitative comments incorporated in subsequent rounds. Survey data were analysed in Statistical Package for the Social Sciences (SPSS) version 25 (IBM Corp., Armonk, NY, USA).

## Results

### Delphi study

#### Participant characteristics

Thirty-two experts participated in Round 1, with 20 completing the final (fourth) round. Participants were 66% male (*n* = 21) and 34% female (*n* = 11), spanning 14 countries across six continents. Most panellists were aged ≥51 years (*n* = 19, 59.4%) with a mean (SD) 22.5 [8] years of professional experience (Supplementary Appendix S2). The panel comprised 24 urologists (75%), 4 nurse practitioners (12.5%) and 4 geriatricians/family physicians (12.5%) in Round 1. Professional representation was similar between the initial and final Delphi rounds (60% urologists, 25% nurse practitioners, 15% geriatricians/family physicians).

#### Consensus outcomes

Consensus was reached on 41 of the 47 proposed UI risk factors. Heart disease, chronic cough, hypertension, poor vision, comorbid conditions and self-perceived health did not reach consensus. All data are presented in Supplementary Appendix S3. Of these 41 other factors, 39 were considered modifiable, and 19 were considered both clinically important and modifiable. These comprised medical conditions (e.g. obesity, constipation and diabetes; *n* = 9), behavioural factors (e.g. smoking, alcohol and caffeine use; *n* = 4), environmental barriers (*n* = 1) and other/functional factors (e.g. impaired mobility, limitation in ADLs, polyuria/nocturnal polyuria; *n* = 5) (Table 1).

**Table 1:** Expert-identified clinically important and modifiable risk factors for urinary incontinence in older men.

	Risk factor/factor group	Domain
1.	Tobacco smoking	Behavioural
2.	Alcohol use	Behavioural
3.	Caffeine intake	Behavioural
4.	Intake of carbonated beverages, citrus, artificial sweeteners	Behavioural
5.	Increased BMI/overweight/obesity	Medical
6.	Constipation	Medical
7.	Diabetes mellitus	Medical
8.	Insomnia/sleep disorder	Medical
9.	Anxiety	Medical
10.	Depression/depressive symptoms	Medical
11.	Urinary tract infection (UTI)/chronic UTI	Medical
12.	Voiding symptoms	Medical
13.	Faecal incontinence	Medical
14.	Environmental barriers (e.g. poor lighting, limited bathroom access, institutional factors)	Environmental
15.	Nocturia/nocturnal polyuria	Other
16.	Poor sleep quality/sleep disturbance	Other
17.	Limitation in physical function/ADL disability	Other
18.	Impaired mobility/immobility	Other
19.	Incontinence less frequent than monthly	Other

These factors were presented to older men to assess willingness and perceived capability.

### Survey

Survey respondents comprised 212 community-dwelling older men (mean age 77.6 ± 7.4 years). Detailed sociodemographic, health perception and UI-related characteristics are provided in Supplementary Appendices S4 and S5.

#### Experience with UI management

Participants reported experience with a range of UI management strategies (Supplementary Appendix S6).

#### Self-efficacy

Mean GSE-UI score was 51.0 ± 35.9, indicating moderate self-efficacy. Confidence was highest in familiar settings and lowest in socially or physically challenging situations (Table 2).

**Table 2:** Distribution of respondents by GSE-UI.

How confident in holding urine	Mean ± SD (*n* = 212)
When at home and have to go to the bathroom	5.03 ± 3.51
When away from home	4.08 ± 3.24
Long enough to get to the bathroom in time during the night	5.61 ± 3.71
For at least 20 minutes when feeling the urge	3.38 ± 3.31
When coughing	4.70 ± 3.77
When sneezing	4.67 ± 3.76
When laughing	5.07 ± 3.80
When nervous	5.17 ± 3.60
When visiting places with difficulty locating the bathroom	3.78 ± 3.20
When going out on social outings without worrying about urine loss	4.13 ± 3.47
Prevent urine loss without relying on pads or protection at home	3.47 ± 3.75
Prevent urine loss without relying on pads or protection when out	2.87 ± 3.61
*Overall GSE-UI score*	*51.0 ± 35.9*

Items were scored from 0 to 10, with higher scores indicating greater confidence. Italic values represent the overall GSE-UI score, calculated as the sum of the individual item scores.

#### Willingness and capability to modify risk factors

Mean willingness and perceived capability scores varied across the 19 expert-identified risk factors (Supplementary Appendices S7A and S8A). The corresponding thresholds used for exploratory quadrant classification were 1.69 for willingness and 1.81 for perceived capability. Mean factor prevalence was 34.9% for the willingness analysis and 33.7% for the perceived capability analysis. These thresholds were used for exploratory quadrant classification of the risk factors.

#### Quadrant-based analysis

Nocturia/nocturnal polyuria, insomnia/sleep disorder, increased BMI/obesity and anxiety exceeded both the prevalence and willingness thresholds (Quadrant I; Supplementary Appendix S7B). Lower-prevalence factors, including constipation, diabetes mellitus, urinary tract infection, voiding symptoms, immobility and physical limitation, demonstrated high willingness (Quadrant III).

For perceived capability (Supplementary Appendix S8B), increased BMI/obesity and anxiety exceeded both the prevalence and capability thresholds (Quadrant I). Lower-prevalence factors with high perceived capability included constipation, diabetes mellitus, urinary tract infection, voiding symptoms, immobility and tobacco smoking (Quadrant III).

In summary, increased BMI/obesity and anxiety exceeded the thresholds for prevalence, willingness and perceived capability, whereas nocturia/nocturnal polyuria and insomnia/sleep disorder exceeded the prevalence and willingness TeX thresholds but not the perceived capability threshold.

#### Prioritisation for intervention development

Nine candidate intervention targets were identified using the quadrant-based framework (Table 3). Four highly prevalent factors (nocturia/nocturnal polyuria, insomnia/sleep disorder, increased BMI/obesity and anxiety) demonstrated high willingness and/or perceived capability (Group A). Five lower-prevalence factors demonstrated high willingness and perceived capability (Group B).

**Table 3:** Patient-reported factors with high willingness and/or capability, stratified by prevalence.

Risk factor	Willingness	Capability	Prevalence
**Group A: High prevalence + high willingness or capability**			
Nocturia/nocturnal polyuria	1.93	1.66	45.8%
Insomnia/sleep disorder	1.87	1.68	38.7%
Increased BMI/obesity	1.74	1.89	41.5%
Anxiety	1.83	1.82	37.3%
**Group B: High willingness + high capability, low prevalence**			
Constipation	1.84	1.91	26.4%
Diabetes mellitus	1.90	1.92	24.1%
UTI/chronic UTI	2.11	2.05	19.8%
Voiding symptoms	2.00	1.86	31.1%
Immobility	1.88	1.83	22.2%

High willingness and perceived capability were defined using the mean factor-specific scores across the 19 risk factors (≥1.69 and ≥1.81, respectively). High prevalence was defined using the mean prevalence across the 19 risk factors for the willingness (34.9%) and capability (33.7%) questionnaires.

#### Associations between self-efficacy and readiness

Willingness and perceived capability were strongly correlated (*r* = 0.81). GSE-UI showed weak positive correlations with willingness (*r* = 0.06) and perceived capability (*r* = 0.17) (Table 4).

**Table 4:** Correlation matrix of GSE-UI, willingness and perceived capability rating scales.

Variable	Spearman’s rank correlation coefficient		
	GSE-UI score	Willingness rating	Perceived capability rating
GSE-UI score	1		
Willingness rating	0.06 (.403)	1	
Capability rating	0.17 (.012^*^)	0.81 (<.001^*^)	1

Spearman’s rank correlation was used due to the ordinal nature of the data. Correlation strength was interpreted as weak (0.1–0.3), moderate (0.3–0.5) or strong (>0.5). *P*-values are shown in parentheses*.*

^*^ *P* < .05.

## Discussion

This study combined expert consensus on clinically important and potentially modifiable UI risk factors with older men’s willingness and perceived capability to modify those factors, providing an empirical approach to prioritising candidate targets for future self-management intervention development. Rather than relying on clinical importance alone, this approach incorporated patient-reported willingness and perceived capability to identify priorities that are both clinically relevant and potentially feasible for older men to address.

### Expert consensus priorities

The Delphi panel identified a broad range of clinically relevant and potentially modifiable factors, reinforcing the multifactorial nature of UI in older men. The inclusion of obesity, constipation, diabetes, sleep disturbances and mobility limitations among the prioritised risk factors underscores the multifactorial nature of UI in older men [3]. These findings are consistent with the recognition that UI commonly coexists with other geriatric conditions and shares overlapping risk factors, reflecting the clustering of geriatric syndromes in older adults [25]. These findings support multidisciplinary approaches to future self-management intervention development rather than focusing solely on urinary symptoms. Comorbidities such as diabetes and depressive symptoms were deemed important and modifiable, consistent with conceptual models linking metabolic and functional contributors to incontinence vulnerability [4]. In contrast, consensus was not reached for heart disease, chronic cough and poor vision, reflecting ongoing uncertainty about the modifiability of some systemic or sensory factors [7].

### Older men’s willingness and capability

Willingness and perceived capability ratings were strongly positively correlated (Table 4), indicating that reported motivation and perceived capability to act often co-occur. Overall, older men reported moderate willingness and perceived capability to modify UI-related risk factors, highest for direct bodily symptoms (e.g. nocturia/nocturnal polyuria, voiding symptoms, constipation) and lowest for lifestyle behaviours such as reducing caffeine or alcohol intake. Weak positive correlations with GSE-UI scores suggest that confidence in managing UI symptoms across different situations is largely distinct from readiness to modify contributing risk factors (Table 4).

Quadrant-based prioritisation identified four highly prevalent factors (nocturia/nocturnal polyuria, insomnia/sleep disorder, increased BMI/obesity and anxiety) together with five lower-prevalence factors demonstrating both high willingness and perceived capability. Lower-prevalence factors with both high willingness and perceived capability, including constipation, diabetes mellitus, urinary tract infection, voiding symptoms and immobility, may represent suitable targets for more individualised interventions.

Common behavioural factors like caffeine intake remained highly prevalent but were associated with lower behavioural readiness, highlighting potential challenges for behaviour change. Lower willingness or perceived capability for behaviours such as reducing caffeine or alcohol intake should not be interpreted as indicating that these behaviours are inherently difficult to modify. Rather, participants’ ratings likely reflect subjective motivation, established habits, perceived benefit or confidence in sustaining behaviour change. Evidence-informed self-management approaches corresponding to these candidate factors are summarised in Box 1 [26–31]. The examples presented in Box 1 illustrate potential self-management approaches reported in the literature. Their effectiveness and long-term sustainability are likely to vary across intervention types, target populations and clinical contexts, highlighting the need for future intervention studies.

**Box 1:** Examples of self-management approaches reported in the literature for the prioritised risk factors.

prioritised risk factor (study findings)	Examples of self-management strategies reported in the literature (varying levels of evidence)
Nocturia/nocturnal polyuria Nocturnal polyuria is strongly linked to fluid redistribution and sodium intake; conservative measures are commonly described in the literature as initial management approaches prior to pharmacologic therapy [26].	**Self-management strategies**: Use of light compression stockings [26] Dietary salt reduction [27] Evening/night-time fluid restriction [26] Late-afternoon or evening leg elevation [28]
Insomnia/sleep disorder Sleep disturbance is associated with increased nocturia and urinary incontinence severity, and non-pharmacologic sleep interventions are recommended as first-line management in older adults [29].	**Self-management strategies:** Sleep hygiene education [29] Regular sleep–wake schedules Limiting evening fluid and caffeine intake [29]
Increased BMI/obesity In older men, higher body mass index and lower physical activity have been associated with increased risk of lower urinary tract symptoms, supporting weight management as a potentially relevant modifiable factor in the literature [30].	**Self-management strategies**: Weight loss through dietary modification Increased physical activity Participation in structured lifestyle programmes [30]
Anxiety Anxiety and psychological distress are associated with urinary symptom severity, and self-management approaches such as mindfulness-based stress reduction have been explored in the literature in reducing urgency incontinence episodes, supporting the use of behavioural strategies to improve coping and symptom perception [31].	**Self-management strategies:** Stress reduction and relaxation techniques Mindfulness-based interventions [31]

The examples presented are intended to illustrate self-management approaches reported in the literature and should not be interpreted as comprehensive management recommendations. Selection of interventions should be individualised according to the underlying cause, comorbidities and patient circumstances.

### Alignment and discordance between clinicians and patients

The principal contribution of this study lies in demonstrating where expert priorities and patient-reported behavioural readiness converge, and where they diverge. Discordance was noted for caffeine and carbonated beverages: experts rated these as important, yet older men reported lower willingness and perceived capability to change, reflecting potential barriers when advice conflicts with established habits. Conversely, high willingness and perceived capability for lower-prevalence factors, including urinary tract infection and voiding symptoms, may help inform future intervention designs aligned with patient priorities. Overall, these findings highlight the importance of integrating clinical priorities with older men’s willingness and perceived capability when designing self-management interventions.

### Self-efficacy and current management practices

Self-efficacy scores indicated moderate confidence, lower in social or unpredictable contexts, consistent with patterns observed in other chronic conditions [14]. This may explain reliance on containment strategies (e.g. protective products), which are easier to implement but do not address underlying symptoms. Behavioural strategies such as timed voiding, pelvic floor exercises and fluid modification are recommended, though uptake depends on perceived benefit and self-efficacy [32].

### Strengths and limitations

A major strength of this study is the integration of international expert consensus and patient-reported behavioural readiness. To our knowledge, this approach has not been previously applied to prioritising intervention targets for UI in older men.

The use of validated instruments and a relatively large community sample strengthens the precision of descriptive estimates. However, the predominantly Caucasian, educated Alberta-based sample may limit transferability to more diverse populations.

The Delphi panel included experts from multiple countries, supporting broad international representation. Accordingly, findings reflect international expert consensus, and may not directly translate to specific healthcare system contexts.

While the older men’s survey used a community-based convenience sampling strategy intended to maximise reach in a population that is often underdiagnosed and underrepresented in clinical datasets, this approach may have resulted in over-representation of individuals with greater healthcare engagement or willingness to disclose symptoms. However, it also enabled inclusion of participants with a wide range of symptom severity and prior management experiences.

Additional limitations include absence of physiotherapists or rehabilitation specialists in the Delphi panel. Attrition occurred across Delphi rounds (32 participants in Round 1 and 20 in Round 4), but predefined participation (>70%) and consensus thresholds (>75%) were maintained throughout, suggesting minimal impact on outcomes [15]. Professional representation remained broadly stable between the initial and final rounds, preserving the multidisciplinary nature of the panel throughout the consensus process.

Supplementary data show participants’ prior experience with UI management strategies (Supplementary Appendix S6); while responses may have been informed by this experience, it does not undermine the assessment of willingness and capability to modify specific risk factors.

### Implications for intervention development and future research

Findings highlight the need for patient-centred, flexible UI interventions that account for both clinical relevance and behavioural readiness. The prioritisation approach presented here may also be applicable to other multifactorial geriatric conditions in which intervention targets must balance clinical importance with patient readiness for behaviour change.

Future self-management intervention development may prioritise high-prevalence factors with high willingness and/or perceived capability (nocturia/nocturnal polyuria, insomnia/sleep disorder, increased BMI/obesity and anxiety), while more individualised approaches may be appropriate for lower-prevalence factors with both high willingness and perceived capability (constipation, diabetes mellitus, urinary tract infection, voiding symptoms and immobility).

### Conclusion

Older men experience a range of UI-related risk factors, with variable readiness to change. Quadrant-based analyses using a structured prioritisation framework integrating prevalence with behavioural readiness (willingness and perceived capability) identified nine candidate risk factors for future self-management intervention development. By integrating expert consensus with behavioural readiness, this study provides an empirical basis for prioritising candidate intervention targets for future self-management research in older men with UI.

## Supplementary Material

Supplementary_materials_afag270

## Data Availability

The data sets generated and/or analysed during the current study are available from the corresponding author on reasonable request.
