# Supplementary material for: Prioritising modifiable risk factors for self-management of urinary incontinence in older men: integrating expert consensus with patient readiness
Source: Age Ageing. 2026 Sep 14;55(9):afag270. doi: 10.1093/ageing/afag270 (PMC13574276; doi:10.1093/ageing/afag270)
Supplement: Supplementary file 1 [file supplementary_materials_afag270.docx]

**Prioritizing Modifiable Risk Factors for Self-Management of Urinary Incontinence in Older Men: Integrating Expert Consensus with Patient Readiness**

**Appendices**

**Appendix 1: Delphi work flow**

Absolute count of panelists by country

|  |  |
| --- | --- |
| Australia | 3 |
| Belgium | 3 |
| Canada | 4 |
| UK | 3 |
| Fiji | 1 |
| France | 1 |
| Hong Kong | 1 |
| India | 2 |
| Iran | 1 |
| Italy | 1 |
| Japan | 1 |
| Netherlands | 2 |
| Nigeria | 1 |
| USA | 8 |

**Appendix 2: Geographical distribution of Delphi panelists**

**Appendix 3: Distribution of risk factors/factor groups by importance in UI development and modifiability (Delphi panel)**

|  | Consensus on both importance and modifiability | Consensus on importance only | Consensus on modifiability only | No consensus on both importance and modifiability |
| --- | --- | --- | --- | --- |
|  | ***Important and modifiable*** | ***Important*** | ***Not modifiable*** | ***No consensus*** |
| 1 | Tobacco smoking | Heart disease | Urethral stricture | Self-perception of health |
| 2 | Alcohol use | Comorbid conditions/increased comorbidity | Kidney disease |  |
|  |  |  | ***Modifiable*** |  |
| 3 | Caffeine intake | Chronic cough | Hypertension |  |
| 4 | Intake of carbonated beverages, citrus, artificial sweeteners | Poor vision |  |  |
| 5 | Increased BMI/overweight/ obesity, Greater waist circumference, Increased fat mass |  |  |  |
| 6 | Constipation/fecal impaction |  |  |  |
| 7 | Nocturia/nocturnal polyuria |  |  |  |
| 8 | Environmental risk factors:  Poor lighting, Cold, Nursing home/ Institutionalization, Early bedtime, Physical restraints, Use of cot-sides/bed rails, Lack of commode, Reliance on draw sheets and pads, Call bells forgotten, Sub-optimal nursing, Commode at improper height, Bathroom distance, Other environmental barriers |  |  |  |
| 9 | Diabetes mellitus |  |  |  |
| 10 | Depression/ Depressive mood/ symptoms |  |  |  |
| 11 | Anxiety |  |  |  |
| 12 | Urinary tract infection (UTI)/Chronic UTI |  |  |  |
| 13 | Insomnia/sleep disorder |  |  |  |
| 14 | *Physical functioning-related factors* Limitation in physical function/ADL disability/low composite physical performance score |  |  |  |
| 15 | *Physical functioning-related factors:* Immobility/Impaired mobility/unable to stand/ Reduced frequency and ease of getting out of the house |  |  |  |
| 16 | Poor sleep quality/ sleep disturbance |  |  |  |
| 17 | Incontinence less frequent than monthly (Rare episodes of trouble controlling pee) |  |  |  |
| 18 | Voiding symptoms (Problems with peeing) |  |  |  |
| 19 | Fecal incontinence (Trouble controlling bowel movements) |  |  |  |
|  | ***Important but not modifiable*** |  |  |  |
| 20 | Brain injury |  |  |  |
| 21 | Prostate cancer, Bladder cancer |  |  |  |
| 22 | Delirium |  |  |  |
| 23 | Demographic risk factors: Age/Increasing age/Advanced age, Occupation, Race/ethnicity, Residential area (urban dwelling) |  |  |  |
| 24 | Cognitive impairment, Dementia |  |  |  |
| 25 | *Nervous system disorders*  Stroke/Prior Stroke, Parkinson's disease, Spinal cord compression/injury/ lesion, Multiple sclerosis, Normal pressure hydrocephalus, Cervical spondylotic myelopathy, Other chronic neurological diseases |  |  |  |
| 26 | BPH/Prostate problems |  |  |  |
| 27 | *Age-related urinary tract functional decline*  Voiding and post-micturition changes: Age-related decline in urethral function, Decreased functional bladder capacity/Increased post void residual volume, Decreased awareness of bladder filling, Decreased efficiency of bladder emptying |  |  |  |
| 28 | Heart Failure |  |  |  |
| 29 | Hip fracture, Foot and ankle problems |  |  |  |
| 30 | *Lower urinary tract-related factors*: Detrusor overactivity, DHIC (Detrusor Hyperactivity with Impaired Contractile Function), Detrusor underactivity/Underactive bladder, Detrusor sphincter dyssynergia (DSD), Atonic bladder |  |  |  |
| 31 | Bladder outlet obstruction |  |  |  |
| 32 | *Medications* Medications/Polypharmacy, Diuretics, CNS depressants, Alpha-antagonists and agonists, Narcotic analgesics, Anticholinergics, Hypnotics/sedatives |  |  |  |
| 33 | Frailty |  |  |  |
| 34 | *Factors related to prostate cancer therapy*  Prostatectomy, Post-radiation therapy/Preoperative radiotherapy, Androgen deprivation therapy for Prostate Ca (PCa), Observation/watchful waiting in PCa, Prostatectomy-related neural injury/ Pudendal nerve injury, Ischemia during surgery, Surgical technique, Sphincter injury, Scar tissue immobilizing the sphincter |  |  |  |
| 35 | *Age-related urinary tract functional decline*  Renal function changes: Decline in renal function, Increase in nocturnal sodium and fluid excretion/Increased nighttime urine production |  |  |  |
|  | ***Not important and not modifiable*** |  |  |  |
| 36 | Low serum testosterone |  |  |  |
| 37 | *Structural factors*  Myosteatosis (Low average total psoas density), Low obturator internus muscle thickness, Short membranous urethral length |  |  |  |
|  | ***Not important and modifiable*** |  |  |  |
| 38 | Physiological risk factor: Decreased maximum grip strength |  |  |  |
| 39 | Chronic diarrhea |  |  |  |

*Reasons for lack of consensus included perceived low modifiability and limited direct association with UI*

**Appendix 4: Sociodemographic characteristics of survey participants**

| **Variable** | **Frequency (n = 212)** | **Percent** |
| --- | --- | --- |
| **Age (year)** |  |  |
| 65-69 | 27 | 12.7 |
| 70-74 | 48 | 22.6 |
| 75-79 | 57 | 26.9 |
| 80-84 | 45 | 21.2 |
| ≥85 | 35 | 16.5 |
| Mean ± SD | 77.6 ± 7.4 |  |
| **Education** |  |  |
| Some high school | 29 | 13.7 |
| Completed high school | 40 | 18.9 |
| Some university/college | 39 | 18.4 |
| Completed a university/college | 104 | 49.1 |
| **Employment status** |  |  |
| Employed | 18 | 8.5 |
| Retired | 194 | 91.5 |
| **Annual household income** |  |  |
| Low | 16 | 7.5 |
| Middle-low | 28 | 13.2 |
| Middle | 89 | 42.0 |
| Middle-high | 45 | 21.2 |
| High | 12 | 5.7 |
| Prefer not to answer | 22 | 10.4 |
| **Race** |  |  |
| Caucasian/White | 194 | 91.5 |
| South Asian | 1 | 0.5 |
| Filipino | 5 | 2.4 |
| Indigenous | 2 | 0.9 |
| Black | 4 | 1.9 |
| Chinese | 3 | 1.4 |
| Others | 3 | 1.4 |
| **Others races** | **n = 3** |  |
| Latino | 1 | 33.3 |
| Vietnamese | 2 | 66.7 |

**Appendix 5: Participants’ Health Perceptions and Urinary Incontinence-Related Characteristics**

| **Variable** | **Frequency (n = 212)** | **Percent** |
| --- | --- | --- |
| **Perception about own health** |  |  |
| Excellent | 16 | 7.5 |
| Very good | 64 | 30.2 |
| Good | 80 | 37.7 |
| Fair | 25 | 11.8 |
| Poor | 9 | 4.2 |
| No response | 18 | 8.5 |
| **Duration of urinary incontinence (years)** |  |  |
| <1 | 9 | 4.2 |
| 1-5 | 61 | 28.8 |
| >5 | 124 | 58.5 |
| No response | 18 | 8.5 |
| **Frequency of urinary incontinence** |  |  |
| ≤1 time/week | 18 | 8.5 |
| 2–3 times/week | 29 | 13.7 |
| 1 time/day | 16 | 7.5 |
| Several times a day | 78 | 36.8 |
| All the time | 53 | 25.0 |
| No response | 18 | 8.5 |
| **Quantity of urine loss each time** |  |  |
| A small amount | 115 | 54.2 |
| A moderate amount | 56 | 26.4 |
| A large amount | 23 | 10.8 |
| No response | 18 | 8.5 |
| **Number of urinary incontinence pads use each day** |  |  |
| None | 54 | 25.5 |
| 1 | 63 | 29.7 |
| 2 | 28 | 13.2 |
| 3 | 13 | 6.1 |
| ≥4 | 36 | 17.0 |
| No response | 18 | 8.5 |
| **Ever seen a doctor about your urinary incontinence** |  |  |
| Yes | 160 | 75.5 |
| No | 52 | 24.5 |
| **Type of urinary incontinence according to doctor** | **n = 160** | **Percent** |
| Stress UI | 9 | 5.6 |
| Urgency UI | 31 | 19.4 |
| Mixed UI | 10 | 6.2 |
| Overflow UI | 1 | 0.6 |
| Functional UI | 18 | 11.3 |
| None | 3 | 1.9 |
| Don’t know | 88 | 55 |

Appendix 6 presents participants’ reported use and perceived effectiveness of various self-management and medical strategies for urinary incontinence. Protective products (pads or special underwear) were most commonly used and rated effective. Behavioral strategies (timed voiding, bladder control exercises, fluid and dietary modifications) were used variably, with many participants reporting limited prior experience or moderate effectiveness. Weight management, medical interventions, and consulting clinicians were similarly variably adopted. Most participants reported quitting smoking as not applicable. These data illustrate the diversity of prior exposure, perceived benefit, and feasibility of different strategies among older men.

**Appendix 6: Older Men’s Experiences with Strategies for Managing Urinary Incontinence**

| **Methods** | **Frequency (n = 212)** | **Percent** |
| --- | --- | --- |
| **Drinking less water** |  |  |
| Have not tried this and would not consider it | 41 | 19.3 |
| Have not tried this but will consider it | 19 | 9.0 |
| Worked well for me | 18 | 8.5 |
| Worked a little bit for me | 58 | 27.4 |
| Did not work for me | 33 | 15.6 |
| Does not apply to me | 43 | 20.3 |
| **Using pads or special underwear** |  |  |
| Have not tried this and would not consider it | 14 | 6.6 |
| Have not tried this but will consider it | 11 | 5.2 |
| Worked well for me | 130 | 61.3 |
| Worked a little bit for me | 26 | 12.3 |
| Did not work for me | 4 | 1.9 |
| Does not apply to me | 27 | 12.7 |
| **Staying home more often** |  |  |
| Have not tried this and would not consider it | 81 | 38.2 |
| Have not tried this but will consider it | 7 | 3.3 |
| Worked well for me | 26 | 12.3 |
| Worked a little bit for me | 22 | 10.4 |
| Did not work for me | 10 | 4.7 |
| Does not apply to me | 66 | 31.1 |
| **Drinking less coffee, tea, or soda** |  |  |
| Have not tried this and would not consider it | 31 | 14.6 |
| Have not tried this but will consider it | 31 | 14.6 |
| Worked well for me | 35 | 16.5 |
| Worked a little bit for me | 49 | 23.1 |
| Did not work for me | 19 | 9.0 |
| Does not apply to me | 47 | 22.2 |
| **Doing exercises for bladder control** |  |  |
| Have not tried this and would not consider it | 30 | 14.2 |
| Have not tried this but will consider it | 64 | 30.2 |
| Worked well for me | 10 | 4.7 |
| Worked a little bit for me | 35 | 16.5 |
| Did not work for me | 46 | 21.7 |
| Does not apply to me | 27 | 12.7 |
| **Going to the bathroom at set times** |  |  |
| Have not tried this and would not consider it | 49 | 23.1 |
| Have not tried this but will consider it | 48 | 22.6 |
| Worked well for me | 21 | 9.9 |
| Worked a little bit for me | 28 | 13.2 |
| Did not work for me | 19 | 9.0 |
| Does not apply to me | 47 | 22.2 |

| **Variable** | **Frequency (n = 212)** | **Percent** |
| --- | --- | --- |
| **Losing weight or keeping a healthy weight** |  |  |
| Have not tried this and would not consider it | 14 | 6.6 |
| Have not tried this but will consider it | 49 | 23.1 |
| Worked well for me | 25 | 11.8 |
| Worked a little bit for me | 35 | 16.5 |
| Did not work for me | 21 | 9.9 |
| Does not apply to me | 68 | 32.1 |
| **Quitting smoking** |  |  |
| Have not tried this and would not consider it | 2 | 0.9 |
| Have not tried this but will consider it | 3 | 1.4 |
| Worked well for me | 0 | 0 |
| Worked a little bit for me | 7 | 3.3 |
| Did not work for me | 1 | 0.5 |
| Does not apply to me | 199 | 93.9 |
| **Avoiding certain foods or drinks that make it worse** |  |  |
| Have not tried this and would not consider it | 25 | 11.8 |
| Have not tried this but will consider it | 54 | 25.5 |
| Worked well for me | 12 | 5.7 |
| Worked a little bit for me | 48 | 22.6 |
| Did not work for me | 25 | 11.8 |
| Does not apply to me | 48 | 22.6 |
| **Taking medicine from a doctor** |  |  |
| Have not tried this and would not consider it | 18 | 8.5 |
| Have not tried this but will consider it | 44 | 20.8 |
| Worked well for me | 20 | 9.4 |
| Worked a little bit for me | 58 | 27.4 |
| Did not work for me | 24 | 11.3 |
| Does not apply to me | 48 | 22.6 |
| **Using a special device** (like clamps or sheaths) |  |  |
| Have not tried this and would not consider it | 72 | 34.0 |
| Have not tried this but will consider it | 40 | 18.9 |
| Worked well for me | 14 | 6.6 |
| Worked a little bit for me | 8 | 3.8 |
| Did not work for me | 8 | 3.8 |
| Does not apply to me | 70 | 33.0 |
| **Talking to a doctor or nurse about it** |  |  |
| Have not tried this and would not consider it | 13 | 6.1 |
| Have not tried this but will consider it | 29 | 13.7 |
| Worked well for me | 43 | 20.3 |
| Worked a little bit for me | 63 | 29.7 |
| Did not work for me | 33 | 15.6 |
| Does not apply to me | 31 | 14.6 |

**Appendix 7A: Distribution of Respondents by the Changes They Do Not or Want to Make**

| **Willingness: changes-do not or want to make** | **Frequency (%) n = 212** | | | | | **Mean Score ± SD** | **Median (IQR)** |
| --- | --- | --- | --- | --- | --- | --- | --- |
|  | **Do not want** | **Might be willing** | **Pretty sure** | **Definitely want to** | **Does not apply** |  |  |
| Tobacco smoking | 3 (1.4) | 0 (0) | 0 (0) | 2 (0.9) | 207 (97.6) | 1.20 ± 1.64 | 0 (0-3) |
| Alcohol use | 26 (12.3) | 30 (14.2) | 6 (2.8) | 6 (2.8) | 144 (67.9) | 0.88 ± 0.91 | 1 (0-1) |
| Caffeine intake | 39 (18.4) | 57 (26.9) | 11 (5.2) | 16 (7.5) | 89 (42.0) | 1.03 ± 0.97 | 1 (0-1) |
| Intake of carbonated beverages | 12 (5.7) | 69 (32.5) | 14 (6.6) | 26 (12.3) | 91 (42.9) | 1.45 ± 0.94 | 1 (1-2) |
| Increased BMI/ overweight/ obesity | 6 (2.8) | 37 (17.5) | 26 (12.3) | 25 (11.8) | 118 (55.9) | 1.74 ± 0.93 | 2 (1-3) |
| Constipation | 9 (4.2) | 17 (8.0) | 18 (8.5) | 24 (11.3) | 144 (67.9) | 1.84 ± 1.06 | 2 (1-3) |
| Nocturia/Nocturnal polyuria | 7 (3.3) | 32 (15.1) | 19 (9.0) | 39 (18.4) | 115 (54.2) | 1.93 ± 1.01 | 2 (1-3) |
| Environmental risk factors: | 10 (4.7) | 17 (8.0) | 4 (1.9) | 6 (2.8) | 175 (82.5) | 1.16 ± 1.01 | 1 (0-2) |
| Diabetes mellitus | 6 (2.8) | 18 (8.5) | 12 (5.7) | 24 (11.3) | 152 (71.7) | 1.90 ± 1.05 | 2 (1-3) |
| Depression/ Depressive mood/ symptoms | 10 (4.7) | 24 (11.3) | 13 (6.1) | 18 (8.5) | 147 (69.3) | 1.60 ± 1.06 | 1 (1-3) |
| Anxiety | 5 (2.4) | 30 (14.2) | 13 (6.1) | 27 (12.7) | 137 (64.6) | 1.83 ± 1.01 | 2 (1-3) |
| Urinary tract infection (UTI)/Chronic UTI | 3 (1.4) | 11 (5.2) | 8 (3.8) | 22 (10.4) | 168 (79.2) | 2.11 ± 1.02 | 2.5 (1-3) |
| Insomnia/sleep disorder | 5 (2.4) | 28 (13.2) | 22 (10.4) | 27 (12.7) | 130 (61.3) | 1.87 ± 0.95 | 2 (1-3) |
| Physical functioning-related factors: Limitation | 9 (4.2) | 12 (5.7) | 12 (5.7) | 27 (12.7) | 152 (71.7) | 1.95 ± 1.13 | 2 (1-3) |
| Physical functioning-related factors: Immobility | 7 (3.3) | 8 (3.8) | 9 (4.2) | 17 (8.0) | 171 (80.7) | 1.88 ± 1.14 | 2 (1-3) |
| Poor sleep quality/ sleep disturbance | 6 (2.8) | 37 (17.5) | 19 (9.0) | 45 (21.2) | 141 (66.5) | 1.96 ± 1.00 | 2 (1-3) |
| Incontinence less frequent than monthly | 12 (5.7) | 16 (7.5) | 12 (5.7) | 31 (14.6) | 141 (66.5) | 1.87 ± 1.16 | 2 (1-3) |
| Voiding symptoms | 5 (2.4) | 20 (9.4) | 13 (6.1) | 30 (14.2) | 144 (67.9) | 2.00 ± 1.02 | 2 (1-3) |
| Fecal incontinence | 6 (2.8) | 13 (6.1) | 12 (5.7) | 17 (8.0) | 164 (77.4) | 1.83 ± 1.06 | 2 (1-3) |
| *Overall score* |  | | | | | *10.6 ± 9.9* | *8 (4-15)* |

*Means calculated among respondents for whom the item was applicable.*

**Appendix 7B: Quadrant Classification of Risk Factors by Willingness and Prevalence**

| **Quadrant I** *(Top Targets)* **High Willingness & High Prevalence (**≥1.69 willingness score, ≥34.9% prevalence)  - Nocturia/Nocturnal polyuria (1.93, 45.8%) - Insomnia/Sleep disorder (1.87, 38.7%) - Increased BMI/Obesity (1.74, 44.3%) - Anxiety (1.83, 35.4%) | **Quadrant II** *(Lower Motivation, Wide Reach)* **Low Willingness & High Prevalence (**<1.69 willingness score, ≥34.9% prevalence)  - Caffeine intake (1.03, 58.0%) - Carbonated beverages (1.45, 57.1%) |
| --- | --- |
| **Quadrant III -** *(Motivated Niche)* **High Willingness & Low Prevalence (**≥1.69 willingness score, <34.9% prevalence)  - Voiding symptoms (2.00, 32.1%) - Poor sleep quality (1.96, 33.5%) - Diabetes mellitus (1.90, 28.3%) - Constipation (1.84, 32.1%) - Physical limitation (1.95, 28.3%) - Immobility (1.88, 19.3%) - Incontinence < monthly (1.87, 33.5%) - UTI / Chronic UTI (2.11, 20.8%) | **Quadrant IV -** *(Lower Priority)* **Low Willingness & Low Prevalence (**<1.69 willingness score, <34.9% prevalence)  - Fecal incontinence (1.83, 22.6%) - Tobacco use (1.20, 2.4%) - Alcohol use (0.88, 32.1%) - Depression (1.60, 30.7%) - Environmental risks (1.16, 17.5%) |

**Appendix 8A: Distribution of Respondents by the Changes that Cannot or Can Be Made**

| **Capability: changes that cannot or can be made** | **Frequency (%) n = 212** | | | | | **Mean Score ± SD** | **Median (IQR)** |
| --- | --- | --- | --- | --- | --- | --- | --- |
|  | **Not able to** | **Might be able** | **Pretty able** | **Definitely** | **Does not apply** |  |  |
| Tobacco smoking | 1 (0.5) | 0 (0) | 0 (0) | 4 (1.9) | 207 (97.6) | 2.40 ± 1.34 | 3 (1-3) |
| Alcohol use | 9 (4.2) | 21 (9.9) | 15 (7.1) | 20 (9.4) | 147 (69.3) | 1.71 ± 1.06 | 2 (1-3) |
| Caffeine intake | 9 (4.2) | 47 (22.2) | 26 (12.3) | 30 (14.2) | 100 (47.2) | 1.69 ± 0.96 | 1.5 (1-3) |
| Intake of carbonated beverages | 5 (2.4) | 50 (23.6) | 22 (10.4) | 33 (15.6) | 102 (48.1) | 1.75 ± 0.94 | 1.5 (1-3) |
| Increased BMI/ overweight/ obesity | 4 (1.9) | 34 (16.0) | 18 (8.5) | 32 (15.1) | 124 (58.5) | 1.89 ± 0.96 | 2 (1-3) |
| Constipation | 6 (2.9) | 17 (8.0) | 9 (4.2) | 24 (11.3) | 156 (73.6) | 1.91 ± 1.08 | 2 (1-3) |
| Nocturia/Nocturnal polyuria | 16 (7.5) | 27 (12.7) | 5 (2.4) | 32 (15.1) | 132 (62.3) | 1.66 ± 1.20 | 1 (1-3) |
| Environmental risk factors: | 5 (2.4) | 12 (5.7) | 6 (2.8) | 9 (4.2) | 180 (84.9) | 1.59 ± 1.07 | 1 (1-3) |
| Diabetes mellitus | 4 (1.9) | 16 (7.5) | 11 (5.2) | 20 (9.4) | 161 (75.9) | 1.92 ± 1.02 | 2 (1-3) |
| Depression/ Depressive mood/ symptoms | 6 (2.8) | 27 (12.7) | 12 (5.7) | 22 (10.4) | 145 (68.4) | 1.75 ± 1.02 | 2 (1-3) |
| Anxiety | 6 (2.8) | 29 (13.7) | 17 (8.0) | 17 (12.7) | 133 (62.7) | 1.82 ± 1.00 | 2 (1-3) |
| Urinary tract infection (UTI)/Chronic UTI | 5 (2.4) | 8 (3.8) | 9 (4.2) | 20 (9.4) | 170 (80.2) | 2.05 ± 1.08 | 2 (1-3) |
| Insomnia/sleep disorder | 10 (4.7) | 29 (13.7) | 15 (7.1) | 24 (11.3) | 134 (63.2) | 1.68 ± 1.05 | 1.5 (1-3) |
| Physical functioning-related factors: Limitation | 10 (4.7) | 13 (6.1) | 21 (9.9) | 20 (9.4) | 148 (69.8) | 1.80 ± 1.06 | 2 (1-3) |
| Physical functioning-related factors: Immobility | 8 (3.8) | 10 (4.7) | 11 (5.2) | 18 (8.5) | 165 (77.8) | 1.83 ± 1.13 | 2 (1-3) |
| Poor sleep quality/ sleep disturbance | 12 (5.7) | 39 (18.4) | 24 (11.3) | 28 (13.2) | 108 (51.4) | 1.66 ± 1.01 | 2 (1-3) |
| Incontinence less frequent than monthly | 9 (4.2) | 21 (9.9) | 13 (6.1) | 23 (10.8) | 146 (68.9) | 1.76 ± 1.08 | 2 (1-3) |
| Voiding symptoms | 6 (2.8) | 24 (11.2) | 9 (4.2) | 27 (12.7) | 146 (68.9) | 1.86 ± 1.07 | 2 (1-3) |
| Fecal incontinence | 6 (2.8) | 12 (5.7) | 6 (2.8) | 13 (6.1) | 175 (82.5) | 1.70 ± 1.13 | 2 (1-3) |
| *Overall score* |  | | | | | *10.4 ± 10.8* | *7 (3-15)* |

*Means calculated among respondents for whom the item was applicable.*

**Appendix 8B: Quadrant Classification of Risk Factors by Capability and Prevalence**

| **Quadrant I** *(Top Targets)* High Capability & High Prevalence  (Capability score ≥ 1.81, Prevalence ≥ 33.7%)  - Increased BMI/Obesity (1.89, 41.5%) - Anxiety (1.82, 37.3%) | **Quadrant II** *(Wide Reach, Limited Ability)* Low Capability & High Prevalence  (Capability < 1.81, Prevalence ≥ 33.7%)  - Caffeine intake (1.69, 52.8%) - Carbonated beverages (1.75, 51.9%) - Nocturia/Nocturnal polyuria (1.66, 37.7%) - Insomnia (1.68, 36.8%) - Poor sleep quality (1.66, 48.6%) |
| --- | --- |
| **Quadrant III** *(Motivated Niche)* High Capability & Low Prevalence (Capability score ≥ 1.81, Prevalence < 33.7%)  - Constipation (1.91, 26.4%) - Diabetes mellitus (1.92, 24.1%) - UTI/Chronic UTI (2.05, 19.8%) - Immobility (1.83, 22.2%) - Voiding symptoms (1.86, 31.1%) - Tobacco smoking (2.40, 2.4%) | **Quadrant IV** *(Lower Priority)* Low Capability & Low Prevalence  (Capability score < 1.81, Prevalence < 33.7%)  - Alcohol use (1.71, 30.7%) - Environmental risks (1.59, 15.1%) - Depression (1.75, 31.6%) - Incontinence < monthly (1.76, 31.1%) - Faecal incontinence (1.70, 17.5%) - Physical limitation (1.80, 30.2%) |
